# Flow Characteristics of Distinctly Viscous Multilayered Intestinal Fluid Motion

**DOI:** 10.1155/2015/515241

**Published:** 2015-05-14

**Authors:** S. K. Pandey, M. K. Chaube, Dharmendra Tripathi

**Affiliations:** ^1^Department of Mathematical Sciences, Indian Institute of Technology, Banaras Hindu University, Varanasi 221005, India; ^2^Department of Mathematics, Echelon Institute of Technology, Faridabad, Haryana 121101, India; ^3^Department of Mechanical Engineering, Manipal University, Jaipur, Rajasthan 303007, India

## Abstract

The goal of this investigation is to study the three layered (core layer, intermediate layer, and peripheral layer) tubular flow of power law fluids with variable viscosity by peristalsis in order to investigate the strength of the role played by an artificially generated intermediate layer to ease constipation. The solution is carried out under the long wavelength and low Reynolds number approximations in the wave frame of reference as the flow is creeping one. The stream functions for each layer such as core layer, intermediate layer, and peripheral layer are determined. The expressions for axial pressure gradient, interfaces, trapping, and reflux limits are obtained. The effects of power law index and viscosities on pressure across one wavelength, mechanical efficiency, and trapping are discussed numerically. It is found that the pressure required to restrain flow rates and the mechanical efficiency increase with the viscosities of the intermediate and peripheral layers as well as with the flow behaviour index. It is observed that the axisymmetric flow in intestines is less prone to constipation than two-dimensional flow and may be more easily overcome with introducing a viscous intermediate layer.

## 1. Introduction

Chyme is a semidigested food mixed with intestinal secrants and is generally non-Newtonian in nature. Its inherent physical behavior is very close to the concept of power-law fluids. A viscous mucuos layer is present as a peripheral layer in the small intestine that acts as a lubricant to safeguard the inner surface of the intestine from the rough contents of the chyme passing through it and secretes enzymes to help digestion. The flow of chyme in intestines is due to* peristalsis* which is a series of rhythmic muscular contractions of the intestinal wall. This is coordinated in such a way that waves appear to start at the point of commencement of oesophagus at the neck and propagate up to the end to culminate after pushing the contents into the stomach through the cardiac sphincter. Further, the flow inside an intestine may be two-dimensional or axisymmetric depending on the state of the content.

In view of huge applications of peristaltic steady and unsteady flow of Newtonian and non-Newtonian fluids, many theoretical studies have been reported in literature time to time. Ng and Ma [1] introduced the Lagrangian approach for peristaltic pumping. Takagi and Balmforth [2, 3] discussed the peristaltic pumping with rigid object and viscous fluids in elastic tube. Dudchenko and Guria [4] studied the self-sustained peristaltic waves. Though these recently published articles are significant contributions in the area of peristalsis, none of them are directly applicable to intestinal flow. Thus, it looks better to concentrate on the most relevant papers.

Brasseur et al. [5] developed two layers peristaltic flow models and studied the effect of peripheral layer viscosity on peristaltic transport through a channel and presented a modified result that a more viscous peripheral layer improves pumping performance while a less viscous peripheral layer degrades the pumping performance. Later on, Rao and Usha [6] extended the previous analytical investigation [5] for a cylindrical flow that yielded similar results. Misra and Pandey [7, 8] investigated peristaltic transport of a non-Newtonian fluid modeled as power-law fluid through a channel as well as a tube in the presence of a peripheral layer and pointed out that flow is enhanced with the peripheral layer viscosity as well as the flow behavior index. They [9] further investigated the peripheral layer effect on a core layer of Casson fluid modelled for blood flow in a channel as well as a tube. Apart from several other results, they concluded that the peripheral layer viscosity favours pumping performance whether the core layer is a power-law fluid or a Casson fluid. Recently, a more general analysis of peristaltic transport with three layers of different viscosities, namely, core, intermediate, and peripheral layers, has been presented by Elshehawey and Gharsseldien [10]. They modeled embryo transfer (the final step of IVF) considering the fluid close to it as the core layer, the culture fluid as intermediate layer, and the intrauterine fluid as peripheral layer. However, this concept of three layers appears appropriate for investigating the role of an artificially generated intermediate layer through medication to intervene constipation. The three-layered peristaltic flow model [10] was further applied by Tripathi [11] to study the axisymmetric blood flow through stenosed arteries by considering red blood cells as the core layer, platelets/white blood cells as the intermediate layer and plasma as the peripheral layer. In a previous attempt [12], we studied such a flow for a channel and concluded that an artificially generated comparatively viscous intermediate layer may help ease constipation. One of the referees was of the opinion that a more appropriate study will be for the tubular geometry. This motivated us to go for this finer quantitative investigation.

In recent years, this mechanism (peristalsis) has been exploited in biomedical engineering, industrial technologies, and toxic waste conveyance in chemical process engineering. Continuous refinements in designs require increasingly more sophisticated models for peristaltic flows using complex working fluids (non-Newtonian, nanotechnological, etc.). The proposed model may be applicable and help investigate similar flows discussed above of biomedical and chemical industries. Therefore, we propose to investigate axisymmetric peristaltic flow of a power-law fluid in three layers through a cylindrical tube. The models [6, 7, 11, 13] will be special cases of this model. Comparative study with previous investigations is made.

This paper is designed as follows.  Section 2 presents the formulation of the model wherein a dimensionless set of governing equations subject to appropriate boundary conditions is derived. Analytical wave form solutions are obtained in Section 3. Interface analysis, pumping characteristic, mechanical efficiency, trapping, and reflux are presented in subsequent sections: from 4 through 8, respectively.  Section 9 provides the detailed numerical results along with interpretations. The final section concludes with our findings.

## 2. Formulation of the Model

**Figure 1:**
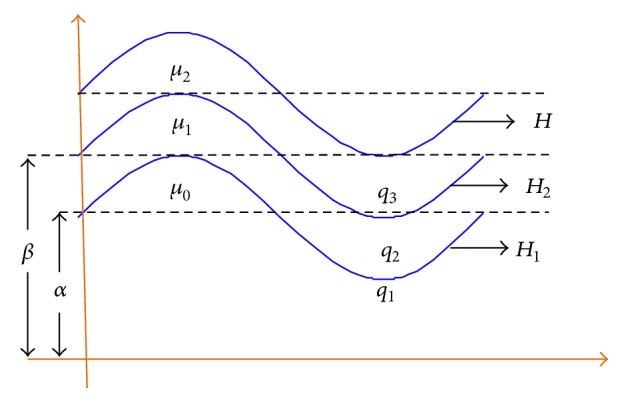
The diagram represents the geometry of the three-layered peristaltic tubular flow. *H*, *H*_2_, *H*_1_ are radial displacement for peripheral, intermediate, and core layers, respectively. *μ*_0_, *μ*_2_, *μ*_1_ and *q*_1_, *q*_2_, *q*_3_ are the viscosities and flow rates in different layers.

We consider the flow of a power-law fluid through a tube in three layers differing in terms of viscosity. As discussed above, the three layers will be called core, intermediate, and peripheral layer. The walls of the tube are considered to be flexible so as to model the small intestine and also because periodic transverse progressive wave trains are to propagate along the walls. Details of the geometry of three-layered (peripheral layer, intermediate layer, and core layer) peristaltic flow region are presented in Figure 1.

The following nondimensional variables are used in the analysis:

(1) z′=zλ,  r′=ra,  t′=ctλ,  u′=uc,v′=vkc,  p′=pan+1μ0λc,  k=aλ,μ¯′=μ¯μ0,  Ψ′=Ψπa2c,  h′=ha,h1′=h1a,  h2′=h2a,  Re=an+1cρλμ0,

where *c*, *λ*, *a*, *n*, and *μ*_0_ represent, respectively, the wave-speed, the wavelength, the radius of the tube, the power-law flow behavior index, and the viscosity parameter of the core fluid. The other parameters are *x*, *y*, *t*, *p*, $\overset{-}{\mu }$, *ψ*, *h*, *h*_1_, and *h*_2_ which represent, respectively, axial and transverse coordinates, time, pressure, viscosity, stream function, and the distance from the centre line of the outer, the intermediate, and the core layer boundary; their primed counterparts are the corresponding quantities in the dimensionless form. *Re* is the Reynolds number.

Using the nondimensional variables and dropping the primes, the equation of the wall may be given by *r* = *H*(*z* − *t*), which may acquire an arbitrary form. Since the three fluids are mutually immiscible and properly conserve the respective masses during the flow, the interfaces between the intermediate and core layers, denoted by *r* = *H*_1_(*z* − *t*), and the peripheral and intermediate layers, denoted by *r* = *H*_2_(*z* − *t*), will be streamlines.

Using the nondimensional variables defined above and considering the long wavelength and low Reynolds number approximations, the governing equations of the flow of a power-law fluid, after dropping the primes, reduce to

(2) $$\begin{array}{c} \frac{\partial p}{\partial z}=\left(\mathrm{sign}\frac{\partial u}{\partial r}\right)\left[\frac{1}{r}\frac{\partial }{\partial r}\left\{r\overset{-}{\mu }{\left|\frac{\partial u}{\partial r}\right|}^{n}\right\}\right],\\ \frac{\partial p}{\partial r}=0. \end{array}$$

The following are the transformations from the laboratory frame to the wave frame:

(3) $$\begin{array}{c} z-t=x,\;\;r=r,\;\;u-1=w,\\ v=v,\;\;p=p, \end{array}$$

where the terms on the left side of the equality sign are in the laboratory frame and those on the right side are in the wave frame; explicitly, *x* and *w* are the axial coordinate and the axial velocity in the wave frame and *u* is the axial velocity in the laboratory frame; other parameters remain invariant in both frames. Accordingly, the stream function *ψ* and the volume flow rate *q* in the wave frame are related to the corresponding terms in the laboratory fame, Ψ being the stream function in the laboratory frame, as given in the following:

(4) $$\begin{array}{c} \psi =\Psi -\frac{r^{2}}{2},\\ q=\bar{Q}-\left(1+\frac{\phi^{2}}{2}\right)=q_{1}+q_{2}+q_{3}, \end{array}$$

where *q*_1_, *q*_2_, and *q*_3_ are the volume flow rates, respectively, in the core, intermediate, and peripheral regions in the wave frame and $\bar{Q}=\int_{0}^{1}Q\;dt$ is the total volume flow rate averaged over a period, henceforth known as the time-averaged flow rate, in the laboratory frame. Consider

(5) $$\begin{array}{c} q_{1}^{*}={\bar{Q}}_{1}-\overset{1}{\underset{0}{\int }}{H_{1}}^{2}\left(x\right)dx=q_{1},\\ q_{2}^{*}={\bar{Q}}_{2}-\overset{1}{\underset{0}{\int }}{H_{2}}^{2}\left(x\right)dx=q_{1}+q_{2}, \end{array}$$

where ${\bar{Q}}_{1}$ and ${\bar{Q}}_{2}$ are the time-averaged flow rates in the core and the intermediate layers, respectively, and *q*_1_^∗^ and *q*_2_^∗^ are parameters defined by the aforementioned expressions used to show the flow-rate relations between the wave and laboratory frames. Equations (2) may be expressed in the wave frame, in terms of stream function *ψ*(*r*, *x*), as follows:

(6) $$\begin{array}{c} \frac{\partial p}{\partial x}=\left[\left(\mathrm{sign}\frac{1}{r}\frac{\partial \psi }{\partial r}\right)\frac{1}{r}\frac{\partial }{\partial r}\left(r\bar{\mu }{\left|\frac{\partial }{\partial r}\left(\frac{1}{r}\frac{\partial \psi }{\partial r}\right)\right|}^{n}\right)\right],\\ \frac{\partial p}{\partial r}=0. \end{array}$$

The variable viscosity is defined as follows:

(7) $$\begin{array}{c} \bar{\mu }=\left\{\begin{array}{cc} 1 & 0\leq r\leq H_{1},\\ \mu_{1} & H_{1}\leq r\leq H_{2},\\ \mu_{2} & H_{2}\leq r\leq H, \end{array}\right. \end{array}$$

and the boundary conditions to be imposed on (6) are

(8) ψ=0,  1rψrr=0, at  r=0,ψr=−H at  r=H,ψ=q2=const at  r=H,ψ=q1∗2=const at  r=H1,ψ=q2∗2=const at  r=H2.

The first boundary condition is that the stream function and the velocity gradient are zero on the symmetry line; the second boundary condition is no-slip condition at the boundary *H* whereas the other three conditions denote mass conservation in the three layers. This further implies that *H*_1_ as well as *H*_2_ is a streamline in the course of the flow.

## 3. Solution in the Wave Frame of Reference

Eliminating the pressure from the differential equations (6), we get

(9) $$\begin{array}{c} \left(\mathrm{sign}\left(\frac{1}{r}\frac{\partial \psi }{\partial r}\right)\right)\frac{\partial }{\partial r}\left[\frac{1}{r}\frac{\partial }{\partial r}\left(r\overset{-}{\mu }{\left|\frac{\partial }{\partial r}\left(\frac{1}{r}\frac{\partial \psi }{\partial r}\right)\right|}^{n}\right)\right]=0. \end{array}$$

Integrating this equation and using the boundary conditions (8), we get

(10) $$\begin{array}{c} \psi =-\frac{1}{2}\left[r^{2}-\left(q+H^{2}\right)\frac{I_{2}}{I_{1}}\right], \end{array}$$

where

(11) $$\begin{array}{c} I_{1}=\overset{H}{\underset{0}{\int }}r\overset{H}{\underset{r}{\int }}{\left(\frac{s}{\bar{\mu }}\right)}^{1/n}ds\;dr,\\ I_{2}=\overset{r}{\underset{0}{\int }}s\overset{H}{\underset{s}{\int }}{\left(\frac{s_{1}}{\bar{\mu }}\right)}^{1/n}ds_{1}\;ds. \end{array}$$

Introducing definition (7), the stream function for the three regions is given by

(12) ψ=−12r2+12r2q+H23n+1n+1 ·1−1μ11/nH1n+1/n+1μ11/n−1μ21/nH2n+1/nmmm+1μ21/nHn+1/n−2n3n+1rn+1/n ×+1μ21/nH3n+1/n−11·1−1μ11/nH13n+1/nmmnmmm+1μ11/n−1μ21/nH23n+1/nmmnmmm+1μ21/nH3n+1/n−1mmmmmmmmmfor  0≤r≤H1,

 

(13) ψ=−12r2+12q+H2 ·1−1μ11/nH13n+1/n+3n+1n+1r2  ·1μ11/n−1μ21/nH2n+1/n+1μ21/nHn+1/nmmmm−2n3n+11μ11/nrn+1/n ×+1μ21/nH3n+1/n−11·1−1μ11/nH13n+1/nmmmmnm+1μ11/n−1μ21/nH23n+1/nmmmnmm+1μ21/nH3n+1/n−1mmmmmmmmfor  H1≤r≤H2,

 

(14) ψ=−12r2+12q+H2 ·1−1μ11/nH13n+1/n+1μ11/n−1μ21/nH23n+1/nmmm+3n+1n+11μ21/nr2mmm1−1μ11/n×Hn+1/n−2n3n+1rn+1/n ×+1μ21/nH3n+1/n−11·1−1μ11/nH13n+1/nmmmnmm+1μ11/n−1μ21/nH23n+1/n    1−1μ11/nH13n+1/n+1μ21/nH3n+1/n−1mmmmmmmmfor  H2≤r≤H.

Moreover, the pressure gradient in view of (12)–(14) is given by

(15) dpdx=−23n+1nn ·q+H2+1μ21/nH3n+1/n−1  ·1−1μ11/nH13n+1/n+1μ11/n−1μ21/nH23n+1/nmmmm1μ11/n−1μ21/n+1μ21/nH3n+1/n−1 ×−q+H2+1μ21/nH3n+1/n−1mmmmm·1−1μ11/nH13n+1/nmmmmmm+1μ11/n−1μ21/nH23n+1/nmmmmmm+1μ21/nH3n+1/n−1n−1.

These results are valid for any arbitrary wave-shape given by *y* = *H*(*x*). A further investigation of the problem will, however, be carried out for a sinusoidal wave form given by

(16) $$\begin{array}{c} H\left(x\right)=1+\phi \sin2\pi x. \end{array}$$

## 4. Interfaces Analysis

Using the boundary conditions (8), we obtain the following equations for the two interfaces:

(17) q1∗+H12q+H2 =1−3n+1n+11μ11/nH13n+1/nmnmm+3n+1n+11μ11/n−1μ21/nH2n+1/nH12mnmm1−3n+1n+11μ11/n+3n+1n+11μ21/nHn+1/nH12  ·1−1μ11/nH13n+1/n+1μ11/n−1μ21/nH23n+1/nmmmm+1μ21/nH3n+1/n−1,

(18) q2∗+H22q+H2 =1−1μ11/nH13n+1/n+1μ11/n−3n+1n+11μ21/nmmmm· H23n+1/n+3n+1n+11μ21/nHn+1/nH22  ·1−1μ11/nH13n+1/n+1μ11/n−1μ21/nmnmmm· H23n+1/n+1μ21/nH3n+1/n−1.

Equations (17) and (18) will be simultaneously solved numerically to evaluate *H*_1_ and *H*_2_ as a function of *x* (see Appendix for the details of solution).

## 5. The Pumping Characteristic

Integrating the axial pressure gradient with respect to *z*, over one wavelength, we get

(19) Δp=−23n+1nn ·∫01γxq+H2H3n+1/n−γxq+H2H3n+1/nn−1dx,

where

(20) γx=1−1μ11/nH13n+1/nH3n+1/n+1μ11/n−1μ21/n  ·H23n+1/nH3n+1/n+1μ21/n−1.

Δ*p* attains its maximum value Δ*p*_0_ when $\bar{Q}=0$. Similarly $\bar{Q}$ reaches ${\bar{Q}}_{0}$, the maximum flow rate, when Δ*p* = 0. Hence Δ*p*_0_ may be explicitly given by

(21) Δp0=−23n+1nn ·∫01γxH2−1−ϕ2/2H3n+1/nmmim·−γxH2−1−ϕ2/2H3n+1/nn−1dx

and ${\bar{Q}}_{0}$ may be evaluated from the implicit relation,

(22) ∫01γxQ¯0+H2−1−ϕ2/2H3n+1/nmm·−γxQ¯0+H2−1−ϕ2/2H3n+1/nn−1dx=0.

## 6. Mechanical Efficiency of Pumping

The mechanical efficiency is defined as the ratio between the average rate per wavelength at which work is done by the moving fluid against a pressure head and the average rate at which the wall does work on the fluid (cf. Shapiro et al. [13]). For a power-law fluid, it will be given by

(23) E=Q¯∫01γxq+H2H3n+1/n−γxq+H2H3n+1/nn−1dx·−γxq+H2H3n+1/nn−1H2−1dx−1   ·∫01γxq+H2H3n+1/n−γxq+H2H3n+1/nn−1mmmmnmm·−γxq+H2H3n+1/nn−1H2−1dx−1.

## 7. Trapping Limits

Trapping is a phenomenon of peristalsis in which an internally circulating bolus of fluid is formed by closed streamlines and this trapped bolus is pushed ahead along with the peristaltic wave (cf. Shapiro et al. [13]). It takes place at high flow rates and large occlusions. Trapping limit is given by the value of $\bar{Q}$ where *ψ* = 0 for *y* > 0. For the given wave information, there is a range of $\bar{Q}$ for trapping to occur. The shape of the trapped region is obtained by setting *ψ* = 0 for *y* ≠ 0 in (12) as follows:

(24) rn+1/n =12nq+H2  ·1−1μ11/nH1n+1/n   ·3n+1q+H2−n+1H12   +1μ11/n−1μ21/nH2n+1/n   ·3n+1q+H2−n+1H22   +1μ21/nHn+1/n3n+1q+H2−n+1H2.

For the occurrence of trapping, we must have *r* ^(*n* + 1)/*n*^ > 0 for some *x*. So, for a real and positive *r* ^(*n* + 1)/*n*^, it is required that both the numerator and denominator be of the same sign. Here, the denominator has its maximum value at *x* = 1/4 and the minimum value at *x* = 3/4. Thus in this case, we have two conditions imposed on $\bar{Q}$ for the existence of trapping. Consider

(25) $$\begin{array}{c} {\bar{Q}}^{-}<\bar{Q}<{\bar{Q}}^{+}, \end{array}$$

where

(26) Q¯−=n+13n+1 ·+1μ21/n1+ϕn+1/n−11−1μ11/nH1max⁡3n+1/n+1μ11/n−1μ21/nH2max⁡3n+1/n+1μ21/n1+ϕn+1/n−1mmnm+1μ21/n1+ϕ3n+1/n   ·1−1μ11/nH1max⁡n+1/nmmimmm+1μ11/n−1μ21/nH2max⁡n+1/nmmmmm+1μ21/n1+ϕn+1/n−1−2ϕ−ϕ22,Q¯+=n+13n+1 ·+ 1μ21/n1−ϕn+1/n−11−1μ11/nH1min⁡3n+1/n   +1μ11/n−1μ21/nH2min⁡3n+1/nmmm+1μ21/n1−ϕ3n+1/nmnm·1−1μ11/nH1min⁡n+1/nmmimmm+1μ11/n−1μ21/nH2min⁡n+1/nmmmmm+ 1μ21/n1−ϕn+1/n−1+2ϕ−ϕ22.

*H*_1max⁡_, *H*_2max⁡_, *H*_1min⁡_, and *H*_2min⁡_ are the values of the interfaces at *x* = 1/4 and *x* = 3/4, respectively. For *n* = 1, the results reduce to the results of Tripathi [11] for three-layered peristaltic viscous flow. For *μ*_1_ = 1, the results reduce to those reported by Misra and Pandey [8] for two-layered power-law flow, which, for *n* = 1, further reduce to those presented by Rao and Usha [6] for two-layered Newtonian flow. The results of Shapiro et al. [13] model can be obtained by substituting *μ*_1_ = 1, *μ*_2_ = 1, and *n* = 1 in presented model.

## 8. Reflux Limit

Reflux is another phenomenon concerning peristalsis in which some fluid particles, on the average, move in a direction opposite to the net flow (cf. Shapiro et al. [13]). The volume flow rate *Q*_*ψ*_(*x*) corresponding to a particle at a position *z* and time *t* in the laboratory frame, and on the streamline *ψ* in the wave frame, is given as

(27) $$\begin{array}{c} Q_{\psi }\left(x\right)=2\psi +r^{2}\left(\psi ;x\right). \end{array}$$

On averaging over one cycle, this becomes

(28) $$\begin{array}{c} {\bar{Q}}_{\psi }\left(x\right)=2\psi +\overset{1}{\underset{0}{\int }}r^{2}\left(\psi ;x\right)dx. \end{array}$$

For evaluating the reflux limit, we expand ${\bar{Q}}_{\psi }\left(x\right)$ in a power series in terms of a small parameter *ε* about the wall, where

(29) $$\begin{array}{c} \varepsilon =\psi -\frac{q}{2}, \end{array}$$

and use the reflux condition

(30) $$\begin{array}{c} \frac{{\bar{Q}}_{\psi }}{\bar{Q}}>1\;\text{as}\;\varepsilon ⟶0. \end{array}$$

The coefficients of the first two terms in the expansion of *r* ^2^(*ψ*; *x*), that is,

(31) $$\begin{array}{c} r^{2}=H^{2}+a_{1}\varepsilon +a_{2}\varepsilon^{2}+\cdots , \end{array}$$

are obtained by using (14) and given by

(32) $$\begin{array}{c} a_{1}=-2,\\ a_{2}=-\frac{4\left(q+H^{2}\right)}{H^{4}\mu_{2}^{1/n}}\gamma \left(x\right). \end{array}$$

Performing the integration in (28) to the second order and using condition (30), we obtain the condition for the occurrence of reflux as follows:

(33) ∫01Q¯−1−ϕ22+H2H1−n/n+1μ21/nH3n+1/n−1   ·1−1μ11/nH13n+1/nmmimm+1μ11/n−μ21/nH23n+1/nmmmim+1μ21/nH3n+1/n−1dx <0.

## 9. Numerical Results and Discussion

We have carried out numerical calculations and plotted graphs to observe the effects of the viscosities of the intermediate and peripheral layers and the flow behaviour index on pumping, mechanical efficiency, trapping limits, and reflux limit.

**Figure 2:**
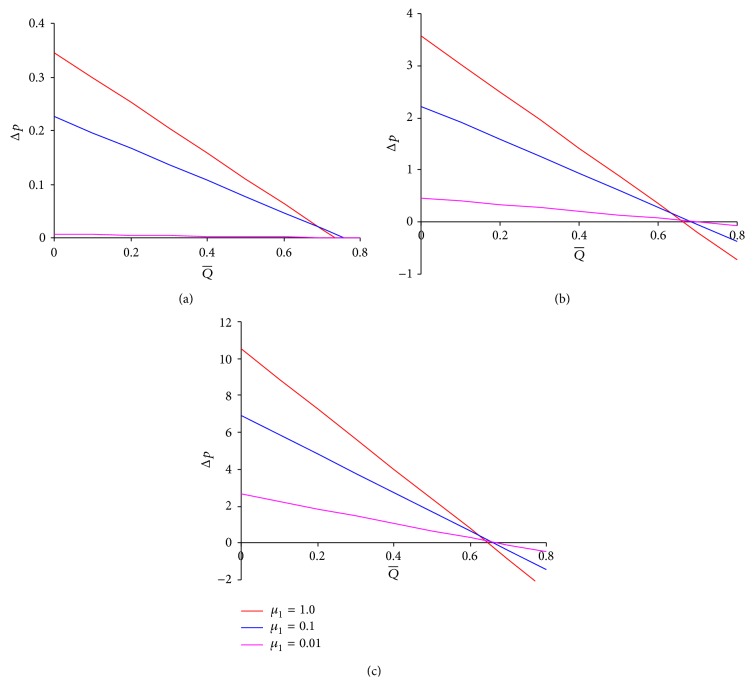
Pressure difference across one wavelength versus time-averaged volume flow rate for different values of *μ*_1_ at *ϕ* = 0.5, *α* = 0.7, *β* = 0.9, and *μ*_2_ = 0.1 for (a) *n* = 0.5, (b) *n* = 1.0, and (c) *n* = 1.5.

**Figure 3:**
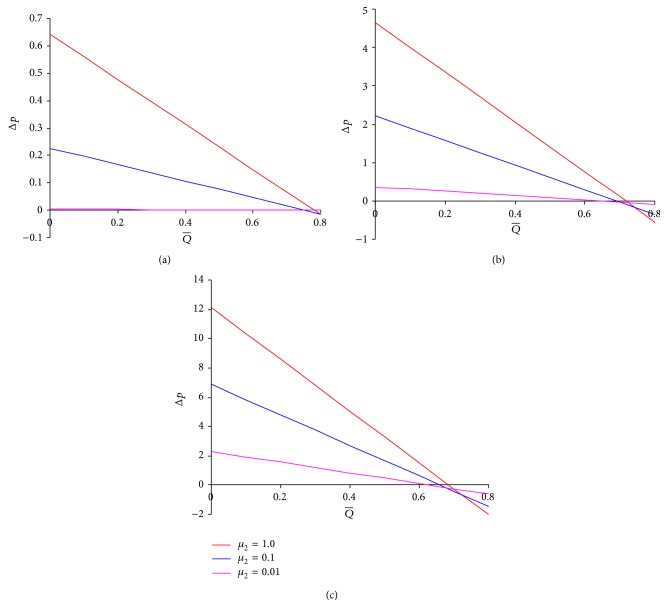
Pressure difference across one wavelength versus time-averaged volume flow rate for different values of *μ*_2_ at *ϕ* = 0.5, *α* = 0.7, *β* = 0.9, and *μ*_1_ = 0.1 for (a) *n* = 0.5, (b) *n* = 1.0, and (c) *n* = 1.5.

**Figure 4:**
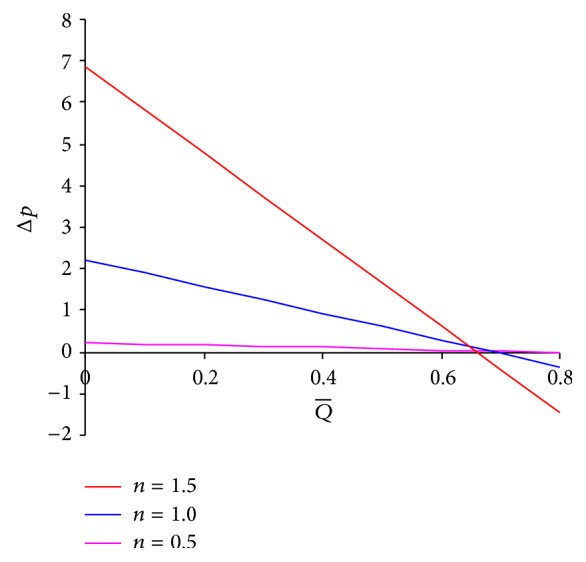
Pressure difference across one wavelength versus averaged volume flow rate for different values of *n* at *ϕ* = 0.5, *α* = 0.7, *β* = 0.9, *μ*_1_ = 0.1, and *μ*_2_ = 0.1.

Figures 2(a)–2(c) are based on (15) and are plotted between the pressure difference across one wavelength (Δ*p*) and the time-averaged flow rate ($\bar{Q}$) to observe the impact of variation of the intermediate layer viscosity *μ*_1_. The relation is linear and the maximum flow rate ${\bar{Q}}_{0}$, which is dependent on *μ*_1_, is achieved when Δ*p* is zero. However, for a fixed *μ*_2_, increase of many folds in *μ*_1_ brings about only a slight cut in ${\bar{Q}}_{0}$ irrespective of the fluid being pseudoplastic (*n* = 0.5), Newtonian (*n* = 1.0), or dilatant (*n* = 1.5) (see Figures 2(a)–2(c)). But a more interesting observation is that the pressure required to restrain flow rates has to increase by a large magnitude if *μ*_1_ is increased even without any variation in *μ*_2_. In Figures 2(a)–2(c), the curves for *μ*_1_ = 1.0 show the results for two-layered peristaltic power-law fluid flow which are similar to results obtained by Misra and Pandey [8]. The results in Figure 2(c) are verified with the results (three-layered peristaltic viscous flow) of Tripathi [11]. Figures 3(a)–3(c) are plotted to examine the influence of the outer (peripheral) layer viscosity on the pressure versus time-averaged flow rate relation. It is noticed that for all the values of flow behavior index, unlike *μ*_1_ an increase in the viscosity *μ*_2_ of the outer layer causes a slight addition to the maximum flow rates. However, on the pressure-flow rate relation *μ*_1_ and *μ*_2_ both have similar effects. The influence of the flow behaviour index *n* on the pressure-flow rate relation is qualitatively similar to that of intermediate layer viscosity *μ*_1_ (cf.  Figure 4).

**Figure 5:**
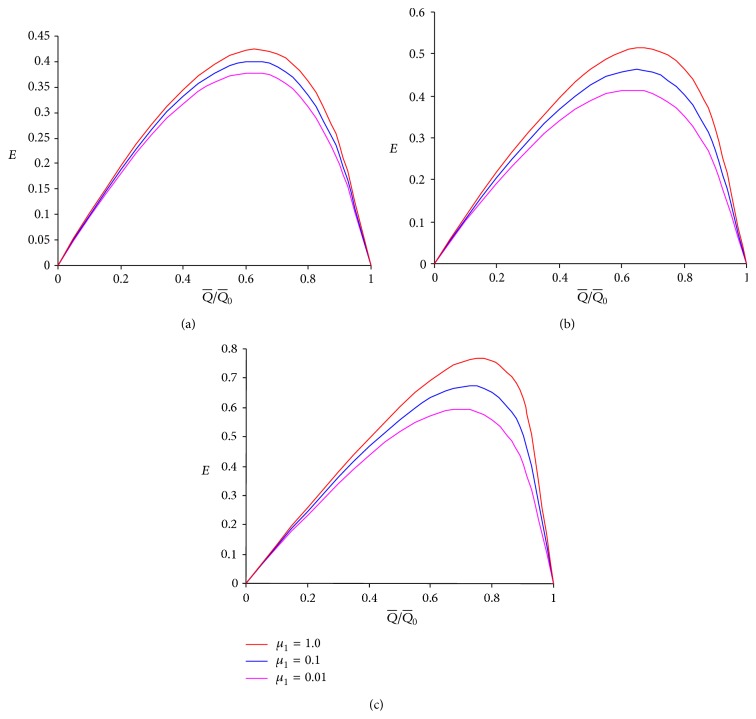
Mechanical efficiency versus ratio of the time-averaged volume flow rate and the maximum time-averaged volume flow rate for different *μ*_1_ at *ϕ* = 0.5, *α* = 0.7, *β* = 0.9, and *μ*_2_ = 0.1 for (a) *n* = 0.5, (b) *n* = 1.0, and (c) *n* = 1.5.

**Figure 6:**
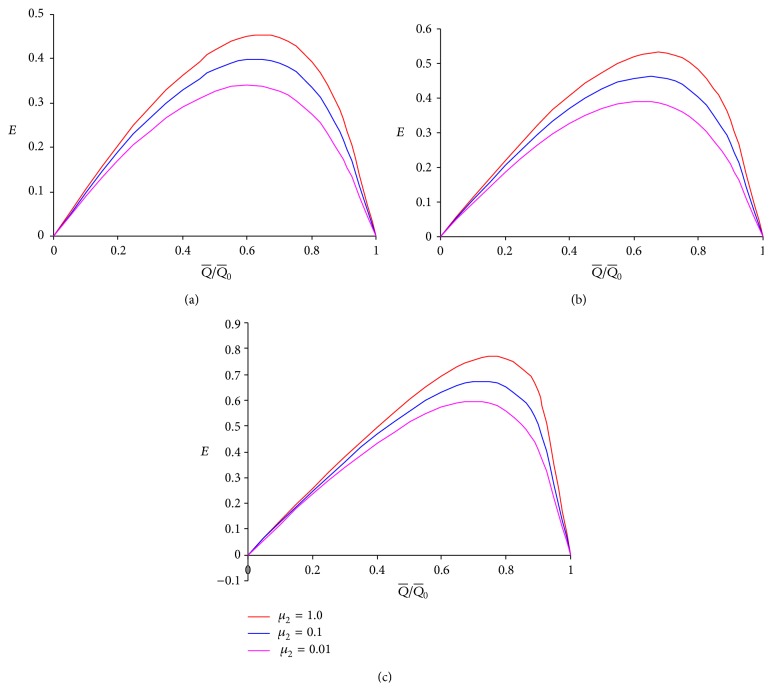
Mechanical efficiency versus ratio of averaged volume flow rate and maximum averaged volume flow rate with the different values of *μ*_2_ at *ϕ* = 0.5, *α* = 0.7, *β* = 0.9, and *μ*_1_ = 0.1 for fixed values of *n* as follows: (a) *n* = 0.5, (b) *n* = 1.0, and (c) *n* = 1.5.

**Figure 7:**
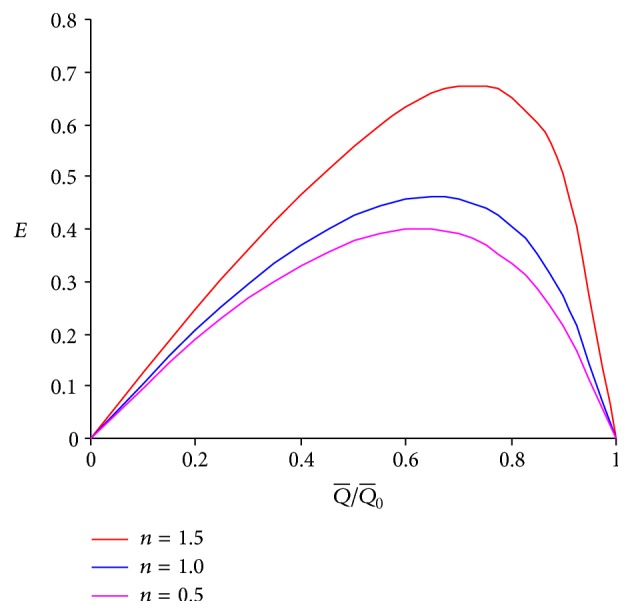
Mechanical efficiency versus ratio of averaged volume flow rate and maximum averaged volume flow rate for different values of *n* at *ϕ* = 0.5, *α* = 0.7, *β* = 0.9, *μ*_1_ = 0.1, and *μ*_2_ = 0.1.

To learn the effects of viscosities in different layers and power-law fluid index on the mechanical efficiency, graphs (Figures 5–7) between mechanical efficiency and the ratio of averaged flow rate and maximum averaged flow rate are plotted based on (23). The relation between them is found to be nonlinear. An observation of Figures 5(a)–5(c) reveals that the pumping performance improves for all values of *n* when the viscosity *μ*_1_ of the intermediate layer is increased. This too favours the earlier conclusion that flow rate is enhanced for larger viscosity of the intermediate layer. The outer layer viscosity *μ*_2_ too is found to improve the pumping performance with an increase in its magnitude (cf. Figures 6(a)–6(c)). The results of Figures 6 and 7 agree with the previous results [8, 11]. The pumping efficiency is found to increase with the flow behavior index (*n*) for fixed viscosities of the outer and the intermediate layers. Thus, peristaltic pumping with a pseudoplastic fluid is less efficient than that with a fluid (cf.  Figure 7).

**Figure 8:**
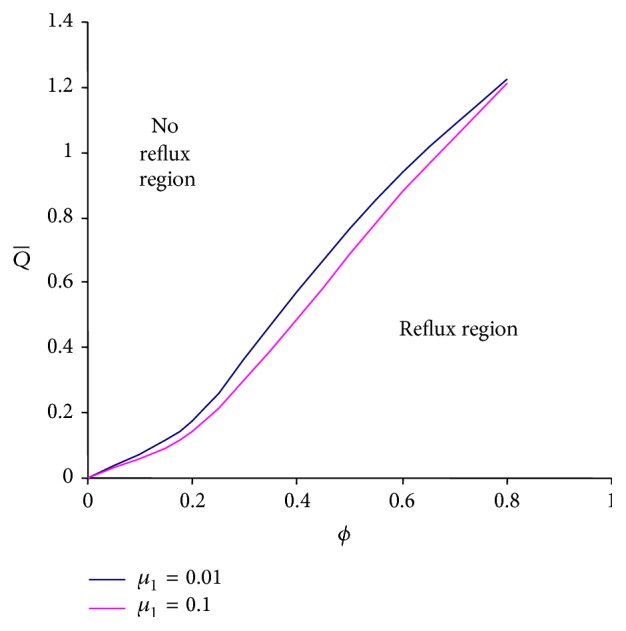
Averaged flow rate versus amplitude ratio with the different values of *μ*_1_ at *ϕ* = 0.5, *α* = 0.7, *β* = 0.9, *μ*_2_ = 0.1, and *n* = 0.5.

**Figure 9:**
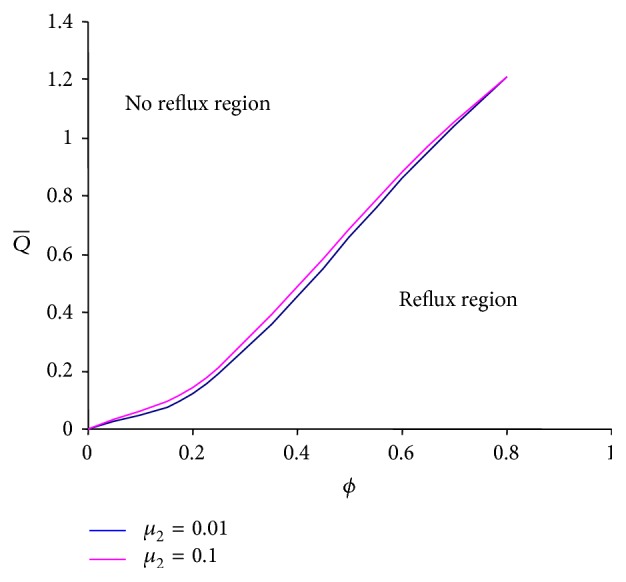
Averaged flow rate versus amplitude ratio with the different values of *μ*_2_ at *ϕ* = 0.5, *α* = 0.7, *β* = 0.9, *μ*_1_ = 0.1, and *n* = 0.5.

Reflux is an inherent phenomenon of peristaltic pumping which is also named reversal flow. To discuss the reflux phenomenon under the effects of viscosities of both layers, Figures 8 and 9 are drawn between the amplitude ratio and averaged flow rate based on (33). It is observed that reflux can occur in a larger region if the intermediate layer viscosity is increased (Figure 8). The outer layer viscosity has a similar effect but the change observed is insignificant (Figure 9).

**Figure 10:**
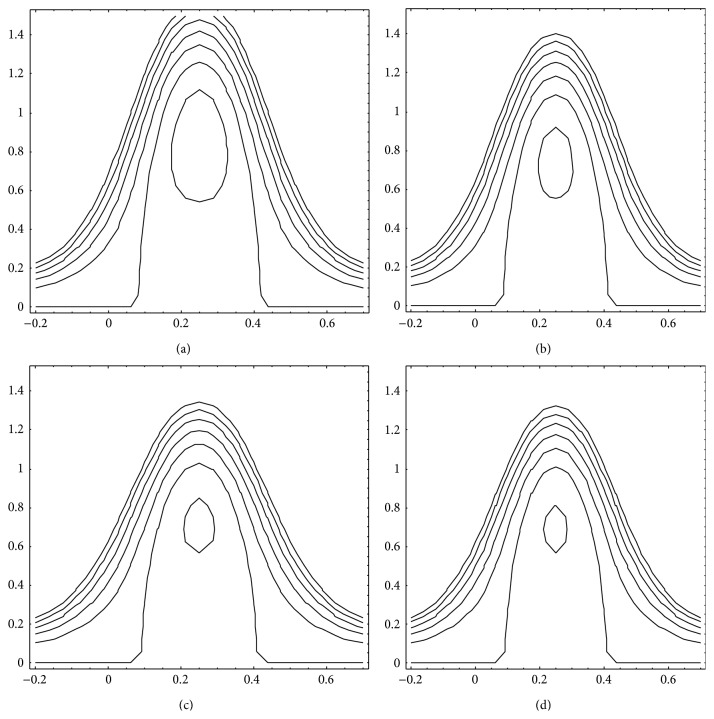
Streamlines for $\bar{Q}=0.7$, *ϕ* = 0.5, *α* = 0.7, *β* = 0.9, *μ*_2_ = 0.02, and *n* = 1.5. (a) *μ*_1_ = 0.02, (b) *μ*_1_ = 0.05, (c) *μ*_1_ = 0.08, and (d) *μ*_1_ = 0.1.

**Figure 11 fig11:**
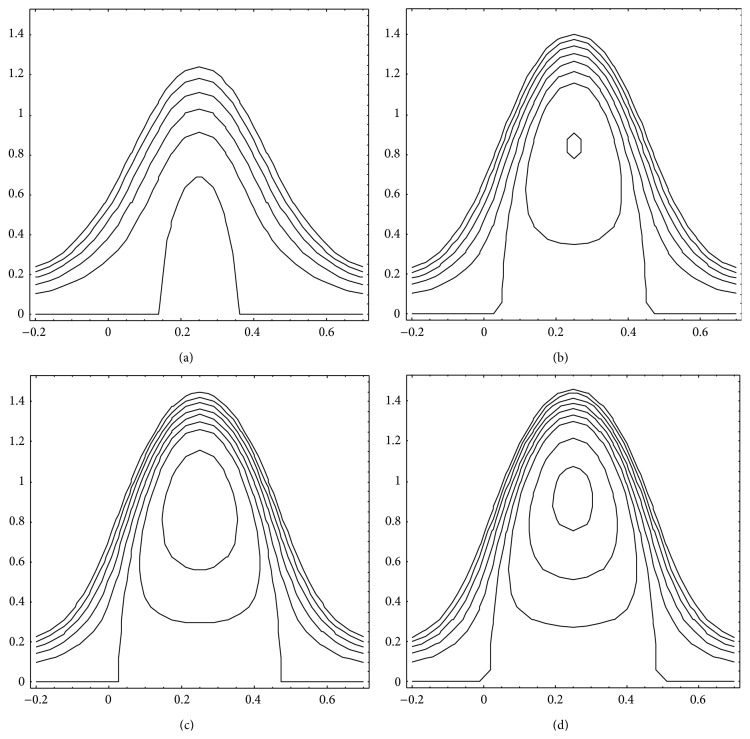
Streamlines for $\bar{Q}=0.7$, *ϕ* = 0.5, *α* = 0.7, *β* = 0.9, *μ*_1_ = 0.1, and *n* = 1.5. (a) *μ*_2_ = 0.01, (b) *μ*_2_ = 0.05, (c) *μ*_2_ = 0.1, and (d) *μ*_2_ = 0.15.

**Figure 12:**
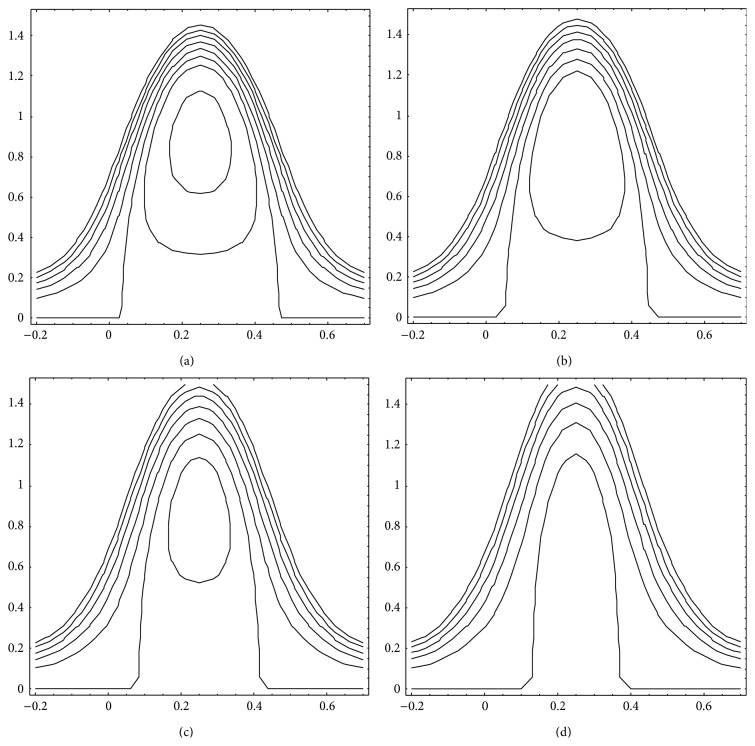
Streamlines for $\bar{Q}=0.7$, *ϕ* = 0.5, *α* = 0.7, *β* = 0.9, *μ*_1_ = 0.1, and *μ*_2_ = 0.1. (a) *n* = 1.4, (b) *n* = 1.2, (c) *n* = 1, and (d) *n* = 0.8.

We have determined the trapping limits based on (26) for different viscosities of the intermediate and peripheral layers and also the flow behaviour index to investigate their effects. It is noticed that when *μ*_1_ varies from 0.01 to 1.0 for the fixed values of *μ*_2_ at 0.1 and *n* at 0.5, ${\bar{Q}}^{-}$ rises from 0.69 to 0.95 and ${\bar{Q}}^{+}$ from 0.95 to 1.1, which means both the lower and the upper trapping limits increase with *μ*_1_, the intermediate layer viscosity. By setting *μ*_1_= 0.1 and *n* = 0.5, if *μ*_2_ is varied from 0.01 to 1.0, ${\bar{Q}}^{-}$ shifts from 0.98 to 0.68 and ${\bar{Q}}^{+}$from 1.1 to 0.95 showing that both the lower and the upper trapping limits decrease with *μ*_2_. When at the fixed values *μ*_1_ = 0.1 and *μ*_2_ = 0.1, *n* varies from 0.5 to 1.5, ${\bar{Q}}^{-}$ varies from 0.74 to 0.33, and ${\bar{Q}}^{+}$ varies from 1.1 to 1.0 indicating thus that both the lower and the upper trapping limits decline with increasing *n*. Depression of trapping limits is an indication that trapping takes place at lower flow rates whereas elevation of the same limits means trapping occurs at higher flow rates. Moreover, the size of the trapped bolus increases with *μ*_2_ and *n* while it decreases with increasing *μ*_1_ (cf. Figures 10 [Fig fig11]–12).

If the entire flow behaviour is further compared with two-dimensional channel flow [12], it is observed that not only the quantity of flow is more but also even the pressure required to stop the flow is almost ten times, thus indicating that the axisymmetric flow is less prone to constipation and may be more easily overcome with introducing a viscous intermediate layer.

## 10. Conclusions

It is found that the maximum flow rate diminishes when the intermediate layer viscosity and flow behaviour index are increased or the peripheral layer viscosity is decreased. It is observed that the pressure required to restrain flow rates has to increase by a large magnitude if *μ*_1_ is increased even without any variation in *μ*_2_ and vice versa. This revelation may lead to the physical interpretation in view of constipation that though the mucous layer viscosity may not be varied the introduction of a viscous intermediate layer caused by some medicinal intervention may cure constipation.

The mechanical efficiency increases with the viscosities of both the intermediate and the peripheral layers as well as the flow behaviour index. Reflux region shrinks if the viscosity of the intermediate layer is increased or that of the peripheral layer is decreased. Both the lower and the upper trapping limits increase if either the viscosity of the intermediate layer is increased or that of the peripheral layer is decreased. The said limits decline when the flow behaviour index is raised. Moreover, the size of the trapped bolus increases with the viscosity of the peripheral layer and the flow behaviour index but it decreases when the viscosity of the intermediate layer is increased.

Finally, it is observed that the axisymmetric flow intestine is less prone to constipation than two-dimensional flow and may be more easily overcome with introducing a viscous intermediate layer.

## Appendix

Equations (17) and (18) form a system of nonlinear algebraic equations in the variables *H*_1_ and *H*_2_ given by

(A.1) $$\begin{array}{c} A_{1}H_{1}^{\left(5n+1\right)/n}+B_{1}H_{1}^{\left(3n+1\right)/n}+C_{1}\left(H_{2}\right){H^{2}}_{1}+D_{1}\left(H_{2}\right)=0,\\ A_{2}H_{2}^{\left(5n+1\right)/n}+B_{2}H_{2}^{\left(3n+1\right)/n}+C_{2}\left(H_{1}\right){H_{2}}^{2}+D_{2}\left(H_{1}\right)=0, \end{array}$$

where

(A.2) A1=1−1μ11/n,A2=1μ11/n−1μ21/n,B1=q1∗1−1μ11/n−q+H21−3n+1n+11μ11/n,B2=q2∗1μ11/n−1μ21/n  −q+H21μ11/n−3n+1n+11μ21/n,C1H2 =1μ11/n−1μ21/nH23n+1/n   +1μ21/nH3n+1/n−3n+1n+1q+H2   ·1μ11/n−1μ21/nH2n+1/n+1μ21/nHn+1/n,C2H1=1−1μ11/nH13n+1/n  +1μ21/nH3n+1/n−3n+1n+1  ·q+H21μ21/nHn+1/n,D1H2 =q1∗1μ11/n−1μ21/nH23n+1/n+1μ21/nH3n+1/n,D2H1=q2∗−q−H21−1μ11/nH13n+1/n  + q2∗1μ21/nH3n+1/n.

The mean thicknesses of these three layers are taken at *x* = 0 and we assume that the interfaces are *H*_1_(0) = *α*, *H*_2_(0) = *β* (the same at *x* = 1/2, 1). The values of *q*_1_^∗^, *q*_2_^∗^ are related to *α* and *β* in the following way:

(A.3) q1∗=−α2+q+1 ·1−3n+1n+11μ11/nα3n+1/n+1μ11/n−1μ21/nβ3n+1/n+1μ21/n−11−3n+1n+11μ11/nα3n+1/nmmmm+3n+1n+11μ11/n−1μ21/nα2βn+1/nmmmm+3n+1n+11μ21/nα2  ·1−1μ11/nα3n+1/nmmmm1−3n+1n+11μ11/nα3n+1/n+1μ11/n−1μ21/nβ3n+1/n+1μ21/n−1,q2∗=−β2+q+1 ·1−3n+1n+11μ11/nα3n+1/n+1μ11/n−1μ21/nβ3n+1/n+1μ21/n−11−1μ11/nα3n+1/nmmmm+1μ11/n−3n+1n+11μ21/nβ3n+1/nmmmm+3n+1n+11μ21/nβ2  ·1−1μ11/nα3n+1/nmmmm1−3n+1n+11μ11/nα3n+1/n+1μ11/n−1μ21/nβ3n+1/n+1μ21/n−1.

## References

[1] Ng C.-O., Ma Y. (2009). Lagrangian transport induced by peristaltic pumping in a closed channel. *Physical Review E*.

[2] Takagi D., Balmforth N. J. (2011). Peristaltic pumping of rigid objects in an elastic tube. *Journal of Fluid Mechanics*.

[3] Takagi D., Balmforth N. J. (2011). Peristaltic pumping of viscous fluid in an elastic tube. *Journal of Fluid Mechanics*.

[4] Dudchenko O. A., Guria G. T. (2012). Self-sustained peristaltic waves: explicit asymptotic solutions. *Physical Review E*.

[5] Brasseur J. G., Corrsin S., Lu N. Q. (1987). The influence of peripheral layer of different viscosity on peristaltic pumping with Newtonian fluids. *Journal of Fluid Mechanics*.

[6] Rao A. R., Usha S. (1995). Peristaltic transport of two immiscible viscous fluids in a circular tube. *Journal of Fluid Mechanics*.

[7] Misra J. C., Pandey S. K. (1999). Peristaltic transport of a non-Newtonian fluid with a peripheral layer. *International Journal of Engineering Science*.

[8] Misra J. C., Pandey S. K. (2001). Peristaltic flow of a multilayered power-law fluid through a cylindrical tube. *International Journal of Engineering Science*.

[9] Misra J. C., Pandey S. K. (2002). Peristaltic transport of blood in small vessels: study of a mathematical model. *Computers and Mathematics with Applications*.

[10] Elshehawey E. F., Gharsseldien Z. M. (2004). Peristaltic transport of three-layered flow with variable viscosity. *Applied Mathematics and Computation*.

[11] Tripathi D. (2012). A mathematical study on three layered oscillatory blood flow through stenosed arteries. *Journal of Bionic Engineering*.

[12] Pandey S. K., Chaube M. K., Tripathi D. (2011). Peristaltic transport of multilayered power-law fluids with distinct viscosities: a mathematical model for intestinal flows. *Journal of Theoretical Biology*.

[13] Shapiro A. H., Jafferin M. Y., Weinberg S. L. (1969). Peristaltic pumping with long wavelengths at low Reynolds number. *Journal of Fluid Mechanics*.

